# Attitudes and knowledge about weight management among primary care physicians in Israel: a cross-sectional study

**DOI:** 10.1186/s12875-024-02324-5

**Published:** 2024-03-19

**Authors:** Keren Or Unger Freinkel, Ilan Yehoshua, Bar Cohen, Roni Peleg, Limor Adler

**Affiliations:** 1 Ben-Gurion University of the Negev, Beer-Sheva, Israel; 2 Department of Family Medicine, Maccabi Healthcare Services, Tel Aviv University, Hamered 27 St., Tel Aviv, Israel; 3 Department of Family Medicine, Faculty of Medicine, Tel Aviv University, Tel Aviv, Israel

**Keywords:** Weight management, Obesity, Primary care physicians, Attitudes, Knowledge, Pharmaceutical treatment

## Abstract

**Background:**

The prevalence of obesity has been increasing worldwide and is associated with increased risk of morbidity and mortality. Weight management can reduce the risk of complications and improve the quality of life of patients with obesity. This study explored primary care physicians’ (PCPs’) attitudes and knowledge about weight management.

**Methods:**

An anonymous questionnaire was distributed to 400 PCPs between 2020 and 2021. The survey included questions on treatment approaches (pharmaceutical and surgical) and items regarding the respondents’ demographic characteristics. We compared PCPs with low or high proactivity toward weight management. We explored attitudes and knowledge with the chi-square test for categorical variables or the Mann-Whitney test for continuous variables.

**Results:**

A total of 145 PCPs answered our survey (a response rate of 36.25%). More than half (53.8%) of the respondents showed low proactivity toward weight management in their practice. Proactive respondents were more likely to believe that pharmaceutical treatment effectively reduces weight and offered medical and surgical treatment options more frequently to their patients. Lack of knowledge was the most predominant reason for PCPs avoiding offering treatment to their patients, especially in less proactive PCPs (33.3% vs. 5.3%, p-value < 0.001). When comparing different pharmaceutical options, 46.6% of PCPs report they tend to prescribe liraglutide to their patients compared with only 11% who prescribe orlistat and 10.3% who prescribe phentermine (p-value < 0.001).

**Conclusions:**

Many PCPs still do not actively provide obesity treatment despite improved awareness and therapeutic options. PCPs’ proactivity and attitudes are vital to this effort.

**Supplementary Information:**

The online version contains supplementary material available at 10.1186/s12875-024-02324-5.

## Background

Obesity is a chronic disease whose prevalence has increased in recent years and is considered a global epidemic. In 2015, about 108 million children and about 604 million adults worldwide were categorized as obese [1]. In the United States, 41.9% of individuals over 20 are obese, and in those aged 2–19, the prevalence of obesity is 19.7% [2]. A recent report in Israel provided data about the prevalence of overweight and obesity in Israel [3]; 34% of adults between 20 and 64 were classified as overweight, and 25.1% were classified as obese as of 2021. In the same report, the prevalence of overweight among children and adolescents was 19.6% and 29.3%, respectively, and for obesity, 7.7% and 12%, respectively. Compared to earlier data, notably from 2013, a marked increase in rates of obesity was reported [3].

Obesity is associated with type 2 diabetes mellitus, hypertension, dyslipidemia, coronary heart disease, etc. [4, 5]. An increase in body mass index (BMI) directly correlates with an increase in morbidity [6]. Among patients with obesity, weight reduction has been proven to improve health outcomes and decrease morbidity and mortality [7, 8]. Studies have shown that even a slight weight loss can reduce morbidity [9]. Moreover, well-timed intervention can potentially reverse specific morbidities, such as pre-diabetes [10].

Weight loss can be achieved by several means, including nutrition, psychological, behavioral, pharmaceutical, and surgical treatment. Weight loss is aimed to prevent and treat the complications of obesity and improve patients’ quality of life. By clinical standards, a successful outcome constitutes a decrease of more than 5% in body weight, reducing complications and improving quality of life [11]. As a first step, patients should be referred for training on lifestyle alterations, including dietary changes, physical activity, and behavioral changes. However, this option is not feasible for all, and many struggle to maintain it.

In such patients, and particularly in patients with persistent obesity and weight-related morbidity, a pharmaceutical option can be considered. This is intended for patients with a BMI higher than 30 or a BMI between 27 and 29.9 who have comorbidities [12]. In Israel, three agents are approved for obesity treatment - Liraglutide, Phentermine, and Orlistat. The much-discussed Glucagon-like peptide-1 receptor agonist (GLP-1 agonist) Semaglutide (Ozempic) is not officially indicated for obesity in Israel and is therefore not included in this study. An additional option for weight loss is surgical intervention. In Israel, bariatric procedures are usually offered to patients with a BMI of over 40 or over 35 if there are comorbid conditions. This method is highly effective and may even produce weight loss of dozens of kilograms in some patients and significant improvements in comorbid conditions [13–15].

### Knowledge and attitudes of physicians in treating obesity

Due to the risks of obesity and the ensuing importance of treating it, it has been long perceived as a matter to be medically discussed by professionals in the field. Different studies explored physicians’ knowledge and attitudes in treating obesity; A study performed in the United States in 2011 reported a difference between physicians practicing in rural communities and those practicing in urban communities. Additionally, a study conducted in Hungary in 2013 reported that only 50% of primary care physicians (PCPs) were familiar with the criteria for obesity treatment. Factors such as training stage, demography, age, and BMI affected attitudes and selection of therapeutic options [16].

A cross-sectional study performed in 2021 in eight European countries reported that most physicians believed that treating obesity in patients with comorbidities was a top priority [17]. However, most physicians selected a lifestyle change; only 30% added pharmaceutical therapy. The common reasons for underprescribing pharmaceutical options were lack of knowledge and concerns about the safety of such options (41%), believing that pharmaceutical treatment should be prescribed by another specialist physician (12%), and not believing in this therapeutic option (13%). In a thematic analysis regarding weight management discussion with patients, some of the obstacles physicians faced were pessimism about weight loss success and physicians’ feelings of hopelessness and frustration regarding the treatment [18].

An Israeli study from 2002 suggested that only 66% of PCPs knew the indication for prescribing pharmaceutical agents, and only 4% actually recommended such treatment to their patients [19]. This sole study performed in Israel highlights the importance of the current study. In 2002, awareness of obesity was still in its infancy, and pharmaceutical options were few and ill-reputed. In the years since some agents were removed due to safety issues (such as Lorcaserin and Sibutramine), safer and more efficient options were introduced.

As new medications for weight management are being introduced constantly [20, 21], PCPs are handling many patients who seek these treatments but do not always feel sure enough to provide them. This study aimed to investigate the attitudes and knowledge of PCPs to weight management and to characterize proactive PCPs in this area.

## Methods

### Study design and setting

In this cross-sectional descriptive study, we distributed surveys to PCPs throughout Israel. Between 2021 and 2022, we offered 400 PCPs to answer our survey, and 145 physicians filled out the questionnaire (a response rate of 36.25%). Participants were not offered incentives to partake. The study was approved by the ethical committee of MHS (0064-21-MHS). Informed consent was granted by submission of a completed questionnaire.

### Participants

The sample in this study was a convenient sample. We distributed the survey during professional conferences, continuing medical education activity, and online professional forums. Only physicians who actively provided direct patient care during the survey period were invited to participate. The survey was open to both specialists and residents. We did not limit the participation based on the number of weekly hours or years of experience.

### Questionnaire

We could not find a questionnaire suitable for our study’s purpose. We formulated and validated a questionnaire through a face-validity process with five different PCPs. The questionnaire has several parts:

- *Attitudes towards weight management*: Section A, questions 2–8, 11–12 and 16.
- *Agreement of the respondents to clinical scenarios in which they would or would not prescribe pharmaceutical therapy* – Section A, question number 9 (11 options).
- *Knowledge enhancers* – Section A, question 10.
- *Attitudes toward different drug agents* (liraglutide, phentermine, and xenical) – Section A, questions 13–15.
- *Demographic questions* – Section B, questions 1–13.

Burris et al. suggested that a proactive approach to managing health behaviors (in their paper, smoking cessation) includes three dimensions: identify the population, offer treatment, and deliver treatment [22]. This model is also relevant for weight management. We defined a proactive PCP as one that identifies his patients with obesity (weighs his patients, initiates a discussion about the subject), offers treatment for it (lifestyle changes/pharmaceutical/bariatric surgeries), and can deliver the treatment himself (when relevant). Proactivity was based on seven items from the questionnaire (2.6, 3.3, 4–6, 12, and 16), with a score between 0 and 8 (see supplementary material for the English translation of the questionnaire and the score). High proactivity was defined as a score above 4.

### Sample size calculations

In order to describe a phenomenon with an assumed proportion of 25%, a confidence level of 95%, and an acceptable difference of 8%, we needed 145 respondents. To compare two proportions (45% vs. 20%) with a significance level of 5% and 80% power, we needed 124 respondents (62 in each group). Sample size calculation was done using Winpepi.

### Statistical analysis

Survey responses were analyzed using SPSS version 28. Descriptive statistics was used; for categorical variables, numbers, and percentages, and for continuous variables, mean and standard deviation. We used univariate analysis to examine the differences between proactive and non-proactive PCPs and the difference in their attitudes using a chi-square test for categorical variables and Mann-Whitney for continuous variables with the non-parametric distribution. Logistic regression was used for multivariate modeling. For the age and sex variables, we used the ENTER approach, and for all other variables, we used the FORWARD approach.

## Results

### Descriptive statistics

The average age of the respondents was 41.6 (± 10.6), with a median value of 38. Women made up 55.8% of the respondents (82/147). 52% were specialists at various stages. On average, the respondents had 10.6 years of experience (± 11.2) and a median of 6 years. 66.4% were graduates of Israeli universities. 15% of the respondents were independent PCPs (fee for service) (n = 23). Almost 54% (76/145) of respondents showed low proactivity (having a low initiative to treat obesity). We did not find significant differences between highly proactive and less proactive PCPs (Table 1). The Cronbach alpha index of the proactivity scale was 0.504.

**Table 1:** Descriptive statistics of highly proactive PCPs vs. less proactive PCPs

Variables	Highly proactive PCPs (n = 76)	Less Proactive PCPs (n = 69)	P value
Age, mean (± SD)	42.33 (11.3)	40.72 (9.9)	0.442
Gender (male)	36 (49.3%)	27 (39.1%)	0.222
BMI			0.447
Did you try to lose weight?			
Yes	49 (66.2%)	45 (67.2%)	0.905
No	25 (33.8%)	22 (32.8%)	
Measures			
Lifestyle	53 (96.4%)	47 (95.9%)	
Medications	2 (3.6%)	1 (2%)	0.509
Surgeries	0 (0%)	1 (2%)	
Specialty status			
Resident	35 (46.7%)	39 (59.1%)	0.158
GP specialist	33 (44%)	22 (33.3%)	
Other	7 (9.4%)	5 (7.6%)	
Years of experience, mean (± SD)	12.08 (11.5)	9.46 (10.9)	0.086
Type of practice			
Urban	60 (81.1%)	55 (82.1%)	0.877
Rural	14 (18.9%)	12 (17.9%)	
Employment status			
On salary	50 (69.4%)	52 (77.6%)	
Independent	14 (19.4%)	9 (13.4%)	0.540
Combined	8 (11.1%)	6 (9%)	
Practice SES			
Low	11 (15.1%)	14 (20.6%)	
Medium	42 (57.5%)	40 (58.8%)	0.524
High	20 (27.4%)	14 (20.6%)	
Where did you learn?			
Israel	45 (60.8%)	48 (70.6%)	0.221
Other	29 (39.2%)	20 (29.4%)	

### Univariate analysis

In univariate analysis, no statistically significant differences were detected between proactive and non-proactive PCPs, including in age, gender, weight, or personal experience with trying to lose weight. In addition, the type of specialization, years of seniority, type of clinic, employment status, country of study, and socioeconomic status (SES) of the patients did not affect the tendency to be proactive in treating obesity. Only 0.02% (n = 3) of PCPs tried pharmaceutical therapy themselves. In a multivariate analysis, no variable was significantly correlated with proactivity (*age*: OR-1.02, 95 CI 0.98–1.05, p-value-0.377; *Gender, female*: OR-0.63, 95% CI 0.31–1.28, p-value-0.200).

### Attitudes toward weight management

**Fig. 1:**
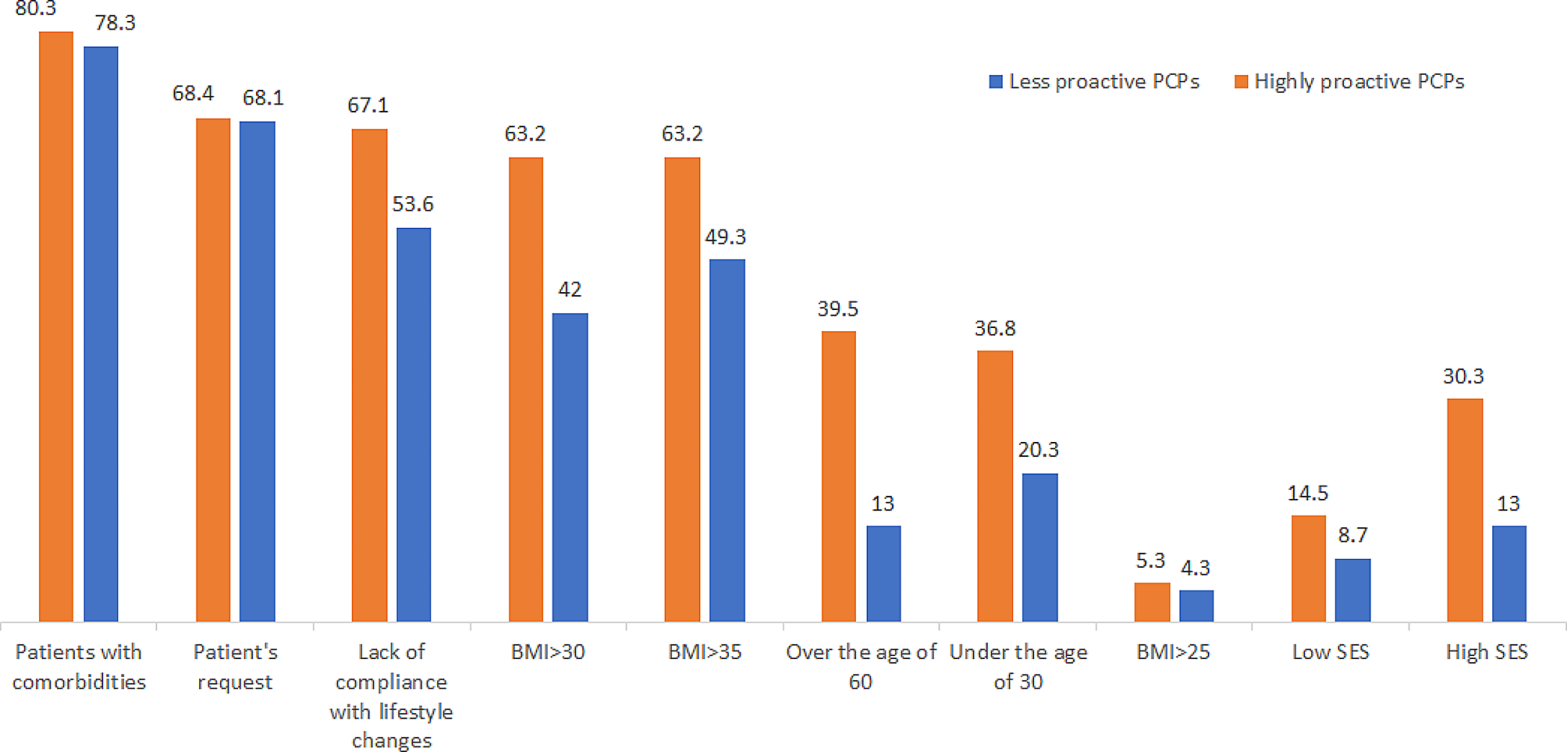
Answers to Q9 – To which of these patients would you offer pharmaceutical treatment for obesity?

We found significant differences in the attitudes toward managing obesity and the proactivity of the physician (Table 2). Proactive PCPs believed that pharmaceutical treatment reduces weight more effectively than non-proactive PCPs (81.6% vs. 53.6%, p-value = < 0.001, respectively). The proactive physicians also offered medical and surgical treatment more frequently to their patients when compared to the non-proactive physicians (65.3% vs. 28.4%, p-value < 0.001 and 13.7% vs. 3%, p-value-0.024, respectively). Proactive PCPs offered medication to a significantly wider variety of patients compared to less-proactive physicians (Fig. 1); patients with a BMI higher than 30 (63.2% vs. 42%, p-value = 0.011), patients who have a low response to lifestyle changes (67.1% vs. 46.4%, p-value = 0.012), patients over the age of 60 (39% vs. 13%, p-value = < 0.001) and young people under the age of 30 who suffer from obesity (36.8% vs. 20.3%, p-value = 0.028). PCPs defined as less proactive claimed that they do not offer pharmaceutical treatment due to the lack of knowledge on the subject (33.3% vs. 5.3%, p-value = < 0.001). Not surprisingly, almost 100% of PCPs in both groups suggested lifestyle modifications for their patients with obesity.

**Table 2:** Comparison of the approaches of proactive PCPs versus non-proactive PCPs in lifestyle treatment/drug treatment / surgical intervention

Variables	Highly proactive PCPs (n = 67)	Less proactive PCPs (n = 78)	P-value
**Q2. To which extent do you agree with the following statements?**			
Lifestyle modification is effective in reducing weight (agree/strongly agree)	56 (81.2%)	63 (82.9%)	0.786
Pharmacological treatment is effective in reducing weight (agree/strongly agree)	62 (81.6%)	37 (53.6%)	< 0.001
Surgical treatment is effective in reducing weight (agree/strongly agree)	54 (71.1%)	42 (60.9%)	0.195
Obesity medication has a high rate of side effects (agree/strongly agree)	20 (26.6%)	16 (23.6)	0.298
Surgical treatment of obesity has significant complications (agree/strongly agree)	12 (15.8%)	6 (8.8%)	0.360
**Q7. If you do not tend to offer pharmaceutical treatment to patients, what are the reasons for it?**			
lack of knowledge	4 (5.3%)	23 (33.3%)	< 0.001
do not know the indication	4 (5.3%)	5 (7.2%)	0.621
	10 (13.2%)	10 (14.5%)	0.816
expensive	11 (14.5%)	12 (17.4%)	0.631
Do not believe in this treatment	8 (10.5%)	11 (15.9%)	0.334
Concern about the side effects	11 (14.5%)	11 (15.9%)	0.806
Unresponsiveness of the patient	13 (17.1%)	9 (13%)	0.496
**Q8. How often do you offer these treatments to your patients? (Always or often vs. rarely or almost never)**			
lifestyle modification	75 (100%)	67 (98.5%)	0.292
pharmacological treatment	49 (65.3%)	19 (28.4%)	< 0.001
surgical treatment	10 (13.7%)	2 (3%)	0.024

As for attitudes regarding improving knowledge and learning about obesity treatment (Table 3), 84% of survey respondents (n = 123) believed that personal experience with patients would improve knowledge and therapeutic approaches. We found that 68% of PCPs offered such treatment at least once a month, and 23% offered it weekly. When asking PCPs what would encourage them to offer more patients treatment for obesity, the most important factors were research that proves the effectiveness of the pharmaceuticals (86.8%) and clear guidelines published by the professional union (82.7%). In contrast, only 12% (n = 18) responded that information published to the general public would affect their approach and treatment of obesity.

**Table 3:** Comparison of the attitudes of proactive PCPs versus non-proactive PCPs regarding improving knowledge and learning about obesity treatment

Variables	Highly proactive PCPs (n = 76)	Less Proactive PCPs (n = 69)	P-value
**Q10. What would add to your knowledge and attitude toward managing obesity? (agree/strongly agree)**			
Personal experience with patients	65 (86.7%)	58 (85.3%)	0.813
Medical journalism	45 (60%)	33 (49.3%)	0.199
Colleague	57 (76%)	49 (72.1%)	0.591
Medical school	27 (37%)	21 (31.8%)	0.522
Conferences and courses	47 (63.5%)	44 (64.7%)	0.882
Public press and television	10 (13.5%)	8 (11.9%)	0.780
**Q11. What would make you offer more medications to patients for treating obesity?**			
Lower price	51 (69.9%)	37 (56.9%)	0.114
Proven by studies	71 (93.4%)	55 (83.3%)	0.058
Peer experience	52 (72.2%)	46 (70.8%)	0.851
Patients demand	41 (53.9%)	26 (40%)	0.098
Better safety profile	60 (78.9%)	52 (78.8%)	0.981
Clear guidelines published by the professional union	62 (82.7%)	58 (87.9%)	0.386

**Fig. 2:**
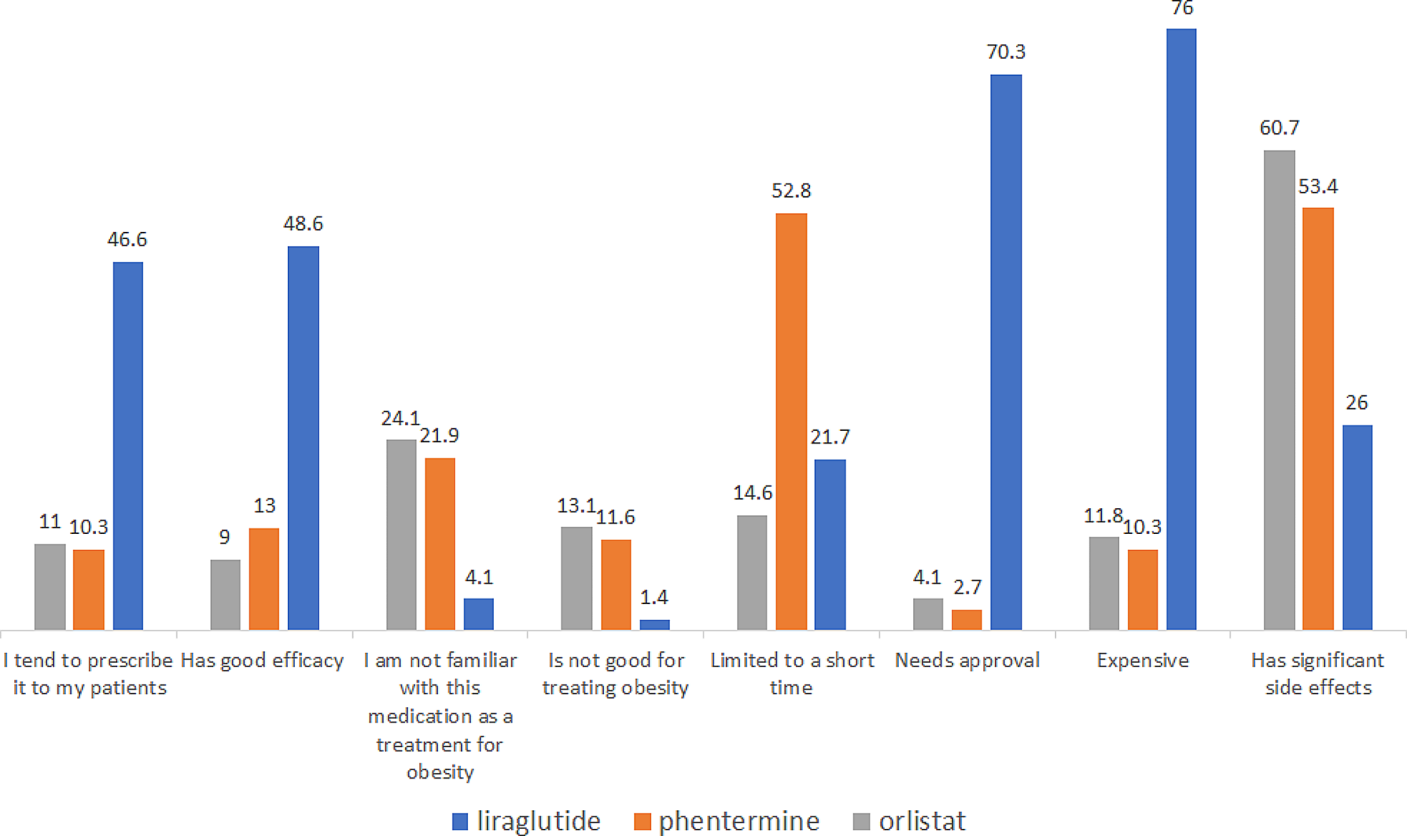
Attitudes and knowledge of PCPs to all weight management medications available in Israel

As for the difference in PCPs’ attitudes to various pharmaceutical agents, statistically significant differences were detected in all the parameters between the three agents tested - liraglutide, phentermine, and orlistat (Fig. 2). Almost half of the PCPs claimed that Liraglutide had good efficacy, compared to 13% and 9% for Phentermine and Orlistat, respectively (p-value < 0.001). The medication used by most PCPs was Liraglutide (46%). In the price category, Liraglutide was perceived as expensive by 76% of PCPs compared to Phentermine (10%) and Orlistat (11%). Orlistat and Phentermine were perceived as having significant side effects (53% and 60%) compared to Liraglutide (26%). Regarding the knowledge of the PCPs, Orlistat (24%) and Phentermine (21%) were considered by more PCPs than Liraglutide as treatments they are not familiar with (4%).

## Discussion

This study examined the current attitudes of PCPs in Israel toward managing obesity. The study provides insight into the factors influencing PCPs’ decisions. PCPs are well familiar with obesity treatments and are willing to see this issue as part of their responsibility. Similar results were observed in studies conducted in Europe and Israel in recent years [17, 19]. Despite the awareness of obesity as a medically relevant issue, only 76% of the respondents thought treating it was their responsibility, and they initiated only half of the conversations on the subject. This is in line with other studies that suggest that although most physicians agree that obesity is a chronic disease, most physicians wait for the patient to broach the subject of weight management [23–25].

Obesity treatment is multilayered, and the first line of treatment, according to the guidelines, is lifestyle modification. As we saw in the study results, almost 100% of PCPs suggested their patients with obesity make lifestyle modifications. Similar results were observed in an Israeli study that examined the attitudes of PCPs in Israel on the subject in 2002 [19]. The similarities between studies conducted more than twenty years apart, with drastically different obesity rates and weight management options, may suggest physicians are comfortable and familiar with this option. When assessing which dietary advice for weight management PCPs tend to give, the most prevalent is reduced caloric intake, but intermittent fasting and ketogenic diet are also quite popular [26].

Additionally, this study examined the familiarity and prescription practices of pharmaceutical options for weight management among PCPs in Israel. We report that most physicians do so on a monthly basis, at least. Almost two-thirds of proactive PCPs offer pharmaceutical options to their patient compared to less proactive PCPs, who offer it to 28%. This may result from their familiarity and confidence in the agents, particularly in liraglutide, a GLP-1 analog. Compared to the Israeli study from 2002, where only 4% of PCPs indicated that they usually prescribe medication for obesity, the US data from 2018 were even lower [27]. This trend may very well result from the trajectory of use and experience. Very early weight loss agents were revealed to be unsafe after they were tested and sometimes marketed; this may have instilled suspicion in physicians looking to safeguard their patients. The case of Liraglutide, however, has allowed physicians to familiarize themselves with the agent as a treatment for diabetes mellitus before its use for other indications. Studies and years of clinical experience have expanded the range of safe and effective therapeutic options, and trends seem to corroborate this. This may also be a consequence of rising obesity rates and concern for associated comorbidities.

As for bariatric surgeries, only 13.7% of proactive PCPs offer this option, compared to even less among less proactive PCPs (3%). This is in line with other studies that suggested that PCPs avoid referring patients to these operations due to overestimation of complications and mortality and the feeling of lack of confidence in treating these patients after the surgery [28].

Comparing the demographic variables that differentiate the groups, no statistically significant differences were found. We assume that there is a knowledge gap between the groups that accounts for the difference between them. PCPs with low proactivity claimed that they do not choose pharmaceutical treatments because of the lack of knowledge. In contrast, proactive PCPs know more about the treatment and its potential drawbacks. Studies show knowledge gaps for weight management options and guidelines and the need of PCPs for more training on obesity [23, 29]. This trend is quite nuanced, as proactive professionals may be more inclined to educate themselves on the matter. Whether the knowledge, or lack thereof, is a consequence of proactivity or its source, it is clear that physicians hesitate to operate where they feel they are not well informed. In such cases, the proactivity of physicians may be encouraged by continued education and discourse, should the need arise.

The finding further highlights that over 80% of PCPs claimed that publishing clear guidelines would make them offer more pharmaceutical treatment to patients. The last time the Israeli Medical Association published such guidelines was in 2003. Such guidelines are almost obsolete, given the new options and the changing trends.

In a Canadian review on improving primary care obesity prevention and management, the authors concluded that a multifactorial approach is needed at the level of education, health policy, and public health; this includes overcoming knowledge gaps and equipping the physician with relevant skills to treat obesity [30]. This means continued education efforts must be done proactively to introduce the full range of therapeutic options. Guidelines should be produced and distributed so physicians feel supported and confident in suggesting pharmaceutical treatment [24].

### Strengths and limitations

This study has several limitations. First, the lack of a validated instrument is a major barrier since the results of this study cannot be compared with similar studies. Second, self-report questionnaires are inherently biased and may be swayed by professional and personal circumstances. A selection bias is possible, as PCPs with a higher awareness of obesity are more likely to participate. The response rate is 36%, which might be viewed as low. However, it is similar to other published PCPs’ surveys. Third, the rapid changes in the field with the introduction and overnight popularity of such agents as Semaglutide are left out of this study. Fourth, obesity management is the work of a multidisciplinary team (physicians, dietitians, psychologists, social workers, etc.). Therefore, future studies should evaluate not only physicians but also other related healthcare workers. Finally, when treating obesity, physicians should try to remain unstigmatized, although, from former studies, this is not the case [31]. This study did not ask about stigmatic attitudes regarding obesity, which may influence attitudes and behavior.

However, this study has several strengths which benefit and enrich the discipline. Primarily, this study addresses physicians’ attitudes, which are sometimes neglected when discussing weight management. While the quest for better health is individual and personal, therapeutic alliances and medical care should and often partake in this process. Additionally, PCPs are a subset of medical professionals who are often the first and most consistent source of medical advice in patients’ lives, rendering them especially valuable in chronic conditions management. The results of this study can be generalized to other primary care practices in developed countries because obesity treatment options (medications and surgeries) are similar. In addition, as seen in the literature, the same obstacles are encountered in many countries when approaching obesity treatment. Yet, cultural and ethnic differences may exist in different countries, which may influence the generalizability of our findings. Lastly, this study explored physicians’ attitudes towards various means of weight loss and management and thus revealed that the aim of weight loss is important in their mind; their obstacles lie in the appropriate mean. This distinction leaves room for intervention and, thus, potentially better care for patients.

## Conclusions

While this study aimed initially to pinpoint the demographic characteristics correlated with increased proactivity in obesity treatment, the resulting findings suggest that the obstacles in such treatment lie not in the individual physician but in the knowledge at their disposal. PCPs feel very confident suggesting lifestyle changes but feel less confident when offering pharmaceutical treatments and even less confident when offering bariatric surgeries to their patients. Our findings suggest that PCPs may be better equipped and empowered to expand the range of therapeutic options for weight management by continuing education and providing them with clear guidelines for weight management.

### Electronic supplementary material

Below is the link to the electronic supplementary material.

Supplementary Material 1

## Data Availability

The datasets generated and analyzed during the current study are not publicly available due to ethical restrictions.

## Abbreviations

BMI: Body Mass Index
GLP-1 agonist: Glucagon-like peptide-1 receptor agonist
PCPs: Primary care physicians
SES: Socioeconomic status
