# Supplementary material for: Attitudes and knowledge about weight management among primary care physicians in Israel: a cross-sectional study
Source: BMC Prim Care. 2024 Mar 19;25:92. doi: 10.1186/s12875-024-02324-5 (PMC10949690; doi:10.1186/s12875-024-02324-5)
Supplement: Supplementary file 1 — Supplementary Material 1 [file 12875_2024_2324_MOESM1_ESM.docx]

**Knowledge and attitudes questionnaire regarding prescribing pharmaceutical treatment for overweight and obesity among community physicians in Israel**

Dear participant,

We are conducting a study concerning obesity treatment among primary care physicians. We would highly appreciate your taking a few minutes to answer the questionnaire. Please note that no identifying information will be disclosed at any point in the study. The questionnaire is filled out anonymously, and the answers will only be used for research purposes. You may choose not to answer the questionnaire or answer the questionnaire partially. By filling in the questionnaire, you give informed consent to participate in this study. Filling out this questionnaire takes approximately five minutes.

Thank you.

Attitudes

1. Do you consent to participate in this study? Yes / No

2. To what extent do you agree with the following statements? (1- strongly disagree, 2-disagree, 3-neutral, 4-agree, 5-strongly agree)

|  | 1 | 2 | 3 | 4 | 5 |
| --- | --- | --- | --- | --- | --- |
| Lifestyle change is effective for weight loss. |  |  |  |  |  |
| Pharmaceutical treatment is effective for weight loss. |  |  |  |  |  |
| Surgical treatment is effective for weight loss. |  |  |  |  |  |
| Pharmaceutical treatment for obesity has many side effects. |  |  |  |  |  |
| Surgical obesity treatment has significant complications. |  |  |  |  |  |
| I believe obesity can be treated. |  |  |  | +1 point | +1 point |

3. Management of obesity is the responsibility of (You may choose more than one option)…

a. The patient

b. A nutritionist

c. The family medicine physician +1 point

d. A physician with another specialty

e. A mental health professional

4. With how many patients have you discussed obesity over the past month? (Please circle your answer)

- 0 patients
- 0-5 patients
- 5-10 patients - +1 point
- over 10 patients - +1 point

5. Who initiated most of these discussions? (Please circle your answer)

- The patient
- The physician - +1 point

6. To how many patients have you suggested pharmaceutical treatment for obesity in the past?

a. I have never suggested pharmaceutical treatment for obesity

b. A few times a year

c. At least once a month - +1 point

d. At least once a week - +1 point

7. If you do not usually suggest pharmaceutical obesity treatment to patients, what are your reasons? (You may choose more than one answer)

a. I do not have enough knowledge on the matter

b. I am not familiar with the indications

c. The medication is not subsidized

d. The medication is expensive

e. I do not believe in this type of treatment

f. I am concerned about the side effects

g. Low adherence of patients with this type of treatment

8. How often do you suggest each of the following treatments to your patients?

|  | Always | Often | Rarely | Almost never |
| --- | --- | --- | --- | --- |
| Lifestyle change | 1 | 2 | 3 | 4 |
| Pharmaceutical treatment | 1 | 2 | 3 | 4 |
| Surgical treatment | 1 | 2 | 3 | 4 |

9. To which of the following patients would you suggest pharmaceutical treatment? (You may choose more than one answer)

a. Overweight (BMI>25)

b. BMI>30

c. BMI>35

d. Obesity with comorbidities

e. Obesity and low adherence to conservative treatment (lifestyle change)

f. Obesity with a failure of conservative treatment

g. Young age (under 30 years of age) and obesity

h. Old age (over 60 years of age) and obesity

i. Low SES

j. High SES

k. By patient’s request

10. What would contribute to your knowledge and therapeutic attitude about obesity? Please note the extent to which you agree with the following statements:

1- strongly disagree, 2- disagree, 3 – neutral, 4- agree, 5 – strongly disagree

|  | 1 | 2 | 3 | 4 | 5 |
| --- | --- | --- | --- | --- | --- |
| Personal experience with my patients in the clinic |  |  |  |  |  |
| Medical journals |  |  |  |  |  |
| Colleagues |  |  |  |  |  |
| Med. School |  |  |  |  |  |
| Medical conferences and courses |  |  |  |  |  |
| Popular media and television |  |  |  |  |  |

11. Which of the following would encourage you to suggest weight loss medications to more patients? Please note the extent to which you agree with the following statements:

1- strongly disagree, 2- disagree, 3 – neutral, 4- agree, 5 – strongly disagree

|  | 1 | 2 | 3 | 4 | 5 |
| --- | --- | --- | --- | --- | --- |
| Medication price decrease |  |  |  |  |  |
| Studies proving medication effectiveness |  |  |  |  |  |
| Colleagues’ experience |  |  |  |  |  |
| Patients demand |  |  |  |  |  |
| Better safety profile |  |  |  |  |  |
| Publication of clear guidelines by professional associations |  |  |  |  |  |

12. How often do you weigh your patients?

a. I do not weigh my patients

b. Once every 3-5 years

c. Once a year

d. Several times a year - +1 point

e. Each visit - +1 point

13. Which of the following is true about Saxenda (Liraglutide) *in your opinion*? You may choose more than one answer.

a. Significant side effects

b. Expensive price

c. Requires approval

d. Limited to short-term use

e. Not suitable for obesity treatment

f. I am not familiar with this medication for obesity treatment

g. It is effective for obesity treatment

h. I prescribe it for my patients

14. Which of the following is true about Razin (Phentermine) *in your opinion*? You may choose more than one answer.

a. Significant side effects

b. Expensive price

c. Requires approval

d. Limited to short-term use

e. Not suitable for obesity treatment

f. I am not familiar with this medication for obesity treatment

g. It is effective for obesity treatment

h. I prescribe it for my patients

15. Which of the following is true about Orlistat (Xenical) *in your opinion*? You may choose more than one answer.

a. Significant side effects

b. Expensive price

c. Requires approval

d. Limited to short-term use

e. Not suitable for obesity treatment

f. I am not familiar with this medication for obesity treatment

g. It is effective for obesity treatment

h. I prescribe it for my patients

16. On a scale of 1 to 10, where 1 is initiative, and 10 is reactive, how would you describe the style of treatment you provide to your patients where obesity is concerned? ________

(1-3 - +2 points, 4-7 - +1 point, 8-10 – 0 points).

Demographic and personal characteristics

1. Age _____

2. Gender (male/female / other)

3. Height ______

4. Weight ______

5. Have you tried to lose weight in the past? Yes / No

6. If you have, what is your primary motivation for losing weight? Health/aesthetics / other (please specify _____)

7. By which means did you try to lose weight? (Please circle your answer)

Lifestyle change / pharmaceutical treatment / surgical treatment

8. Professional status (please circle your answer)

Family medicine resident/family medicine physician / internal medicine physician / general practitioner

9. Years of practicing (post-internship) _____

10. Which type of clinic do you work in?

Rural / Urban

11. What is your employment status?

Hired / independent / both

12. How would you describe the socio-economic status of most patients in your clinic?

High/low/medium

13. Where did you study medicine?

In Israel / abroad

Thank you for your participation!

Mrs. Keren-Or Unger

Dr. Limor Adler

Dr. Ilan Yehoshua

Prof. Roni Peleg

Ms. Bar Cohen
